# Assessment of Self-Care Methods for Acute Dental Pain Among Adults During COVID-19 Dissemination and the Implementation of Teledentistry in Makkah, Saudi Arabia: A Cross-Sectional Study

**DOI:** 10.7759/cureus.33687

**Published:** 2023-01-12

**Authors:** Rahaf K Jarwan, Aseel K Alhindi, Raghad M Iskandar, Shahad O Bashihab, Afnan A Nassar, Shahinaz N Sembawa

**Affiliations:** 1 Dentistry, Umm Al-Qura University, Makkah, SAU; 2 Preventive Dentistry, Umm Al-Qura University, Makkah, SAU

**Keywords:** teledental, self-care, pain management, teledentistry, lockdown, covid-19

## Abstract

Aim: This cross-sectional study aims to explore the prevalence of acute dental pain, different self-care methods done by adults to manage this pain, and the utilization of teledental platforms during the COVID-19 pandemic lockdown period. The study was conducted in Makkah, Saudi Arabia.

Methods: A total of 727 participants answered the study questionnaire, which was distributed as an online survey on different social media platforms. The modified dental pain screening questionnaire (M-DePaQ) was used to obtain dental pain-related characteristics of subjects. Also, self-care and formal-care strategies for pain relief questionnaire for most recent toothache and additional questions developed by the authors related to virtual clinics were used to determine the need for implementation of teledentistry and remote dental consultations. Data was entered in SPSS (IBM Corp., Armonk, NY) and analyzed using descriptive statistics, chi-square test, and binary logistic regression.

Results: Around 39% out of 727 participants experienced dental pain during the COVID-19 Makkah city lockdown; 58% of them reported that the pain was related to a tooth. The majority reported that it was exaggerated with cold beverages and foods [67.1%]. A chi-square association test showed that the age group between 20-30 significantly used the Sehaty (Lean, Riyadh, Saudi Arabia) application more than other age groups. Additionally, women were notably found to use home remedies more frequently than men. Furthermore, individuals with a bachelor’s degree believed more in the importance of having a virtual dental clinic during the periods of lockdown.

Conclusion: During the COVID-19 pandemic, acute dental pain and self-medication were found common among this study population. The practice of self-medication was also documented frequently, therefore, it is important to educate the general population on the proper way to manage dental pain. However, The use of teledentistry services was found uncommon, so to improve patient care especially when accessibility to professional treatment is difficult, the implementation of teledental services need to be considered.

## Introduction

COVID-19 is a severe respiratory syndrome that has resulted in a global concern. The mode of transmission of the virus is usually through direct close contact [1]. The pandemic has led to the closing of most public places. As the cases continued to increase in Saudi Arabia, a complete lockdown and curfew were finally declared [2]. The lockdown has affected all aspects of life in Saudi, including access to healthcare facilities such as professional dental clinics. One of the most common reasons people visit these facilities is dental pain. Pain perception and tolerance differ from one person to another depending on many factors [3]. Acute pain accounts for most dental emergency cases which could be due to a number of different reasons such as trauma, tooth infection, sinus infection and periodontal problems, however, most of the time, the pain is pulpal due to tooth decay [4].

To cope with pain, some people find home remedies convenient and practice “self-medication” which is a term used when a person consumes different drugs or home treatments without a doctor’s consultation. It is usually dependent on self-diagnosis, making it dangerous [3]. One of the most common forms of self-medication is referred to as “Homeopathic Dentistry”. It is known to be a natural but effective treatment alternating prescription drug relief [5]. This is commonly seen in people with low-socioeconomic status who usually find trouble accessing professional dental care, people with dental fear, or those who do not believe in the importance of a dental visit [6]. It was stated that some herbals showed helpful results in treating specific medical conditions. Many medical drugs were derived from plants such as digoxin, quinine, and morphine. Yet, its safety and effectiveness in dentistry are yet to be proven. It has been found that herbal mouthwash was as effective as a placebo for treating mucositis in the oral cavity [7].

A previous study showed it is common for patients to use alternative practices to alleviate toothache; these practices include the use of chemical products such as hydrogen peroxide, battery acid, gasoline, massages, ice, heat, coffee, cloves, and extracting the affected tooth [8]. It had been concluded that self-care strategies have palliative effects until professional dental care is possible [9]. Over-the-counter (OTC) analgesics are also widely used for dental pain such as paracetamol. It is a safe and well-tolerated painkiller. However, its risk of overdosing and hepatotoxicity is often neglected. A previous study found clinical symptoms of hepatotoxicity in people exceeding 4gm per day. Dental pain puts patients at a high risk of accidentally overdosing on paracetamol [10]. A cross-sectional study in India reported that around 75% of the participants did not seek any professional consultation and relied on OTC medications and other treatments [11]. Similar study results showed that 29.4% of the participants who followed self-medication had no interest in recognizing its harmfulness [12].

During the COVID-19 pandemic, people were warned about going to hospitals to prevent the risk of infection [13,14]. This has stopped people from seeking medical care even when required. A study from Italy reported that hospital admissions have decreased by up to 88% in 2020 compared to 2019 and 2018 at the same time of the year [15]. Therefore, the need for a safe but effective way of communication between healthcare providers and patients is required when access to dental care is affected. Teledentistry has been recommended as a suitable alternative [16]. Teledentistry is an evolving system that aims to improve dental care with the use of technology by providing remote consultations. Several studies have described the importance of telemedicine to reduce the risk of infection by COVID-19, especially among dentists [16-17].

Unfortunately, In Saudi Arabia, there has not been a platform for teledentistry yet. Therefore, the present study explores the prevalence of acute dental pain reported during the COVID-19 lockdown in adult Saudi citizens and the different ways people have used it to manage their pain. Furthermore, we explore the utilization and acceptance of teledentistry and remote dental consultations in Saudi Arabia.

## Materials and methods

This study is a cross-sectional survey study. An electronic self-administered questionnaire to assess the self-care methods of Saudi adults for the management of acute dental pain during the COVID-19 lockdown and their subsequent use of teledentistry platforms was distributed through social media platforms during the period of the pandemic from the start of Makkah city lockdown (March 23, 2020) until the end of lockdown (June 21, 2020). The study was explained at the beginning of the questionnaire and respondents’ consent was taken. Ethical approval was obtained from Ethical Research Committee at Umm Al-Qura University (approval number: HAPO-02-K-012-2020-12-514).

Study sample

According to the Saudi Central Department of Statistics and Information, the total population in Makkah region for the latest listing in 2010 was 6,927,477. The total number of adults between 20-60 years was 4,162,724. This study included participants between the ages of 20-40 as well as people in middle adulthood (45-60 ages) from the Makkah region of Saudi Arabia, regardless of their health conditions. The sample size was estimated to be 385 calculated by the Raoasft website according to a 5% error, and 95% confidence interval out of a population size of 4,162,724 [18].

Data collection tool (instrument)

Data has been collected by a self-administered electronic questionnaire that was developed using Google Forms. The questionnaire was structured after consulting relevant studies, including only closed-ended questions; they were also obtained from previous studies and were translated into Arabic. The questionnaire consisted of three parts. First part: socio-demographic data (age, gender, nationality, educational level, long-standing illness, and overall health). Second part: The modified dental pain screening questionnaire (M-DePaQ) which was used to obtain the characteristics of dental pain experienced by the study subjects. Furthermore, a single-item scale ranging from 0 (no pain) to 5 (pain worst) was used to measure participants’ dental pain intensity [12]. Third part: questions about self-care and formal-care strategies for pain relief for most recent toothache [10] and additional questions developed by the authors related to virtual clinics to determine the utilization of teledentistry and remote dental consultations by the study respondents. A pilot study was conducted on 10% of the sample size (73) subjects that were not involved in the main study. Reliability testing was done using Cronbach's alpha test on the pilot answers. The questions were identified and modified according to the results.

The additional questions added were mainly about the "Sehaty" (Lean, Riyadh, Saudi Arabia) application which is a unified app providing several services for individuals, one of which is it provides instant remote medical consultation. This service allows the user to obtain immediate professional help through doctors authorized by the Saudi Ministry of Health [19]. The application is run mainly by medical doctors and no dentists whatsoever. Yet, some participants still chose to seek help through this app.

Statistical analysis

The collected data were statistically analyzed utilizing SPSS software version 27 (IBM Corp., Armonk, NY). The frequency of each question was first obtained. Descriptive statistics were used to describe the data and the chi-square test and binary logistic regression were used to detect associations between demographic variables (age, gender, and educational level) and various inquiries. A significant p-value was set at equal to or less than 0.05.

## Results

Pilot testing

The questionnaire was tested for internal consistency (Cronbach’s alpha=0.86) which indicated good reliability.

Description of demographic data

A total of 727 participants responded to the questionnaire. The mean age of the study population was 34.8 years with the majority of participants in the age range of 20-30 years (51.9%) followed by 31-40 years (16.6%). The least number of participants was above 41 years of age. Gender distribution showed that 62.3% were females and 37.7% were males; 91% of the participants were Saudis, and 73% had a bachelor's degree. Only(13% suffered from a long-standing illness. Furthermore, 65% of the participants thought they had excellent overall health. Demographic data are shown in Table 1.

**Table 1:** Demographic data of participants

		N	Percent
Age	20-30	378	51.9%
	31-40	121	16.6%
	41-50	109	14.9%
	51-60	119	16.3%
Gender	Male	274	37.6%
	Female	453	62.3%
Nationality	Saudi	668	91.8%
	Non-Saudi	59	8.1%
Education	Primary school	3	0.41%
	Elementary school	12	1.6%
	High School	97	13.3%
	Diploma	47	6.4%
	Bachelor	536	73.7%
	Others	32	4.4%
Long-standing illness	Yes	97	13.3%
	No	630	86.6%
Overall health	Excellent	474	65.1%
	Good	218	29.9%
	Fair	35	4.8%

Dental pain intensity and characteristics (M-DePaQ)

In the present study, 280 out of 727 participants experienced dental pain during the COVID-19 Makkah city lockdown. They answered the modified dental pain screening questionnaire (M-DePaQ) to obtain more information on the nature of dental pain for each individual, as seen in Table 2. More than half of them reported that the pain was related to a tooth (58%). A large proportion of the respondents reported experiencing moderate to severe pain (47.9%), causing difficulty in sleeping (45%). The majority reported that the pain was episodic (86.1%) which lasted for 1-3 days (57.5%), radiated to surrounding areas (70.4%), worsened with chewing and eating (79.6%) and when eating or drinking something cold (67.1%).

**Table 2:** The modified dental pain screening questionnaire (M-DePaQ)

		n	%
Did you experience dental pain during Covid-19 Makkah city lock down	Yes	280	38.5%
	No	447	61.4%
Pain in the	Tooth/teeth	162	57.9%
	Gums	62	9.3%
	Both	92	32.9%
Pain for (chronicity of current pain)	1-3 days	161	57.5%
	A week	48	17.1%
	More than 1 week	71	25.4%
Pain is (dental pain intensity scale)	No pain	14	5%
	Mild	89	31.8%
	Moderate – severe	134	47.9%
	Very severe	29	10.4%
	Worst pain possible	14	5%
Pain has been (pattern of current dental pain)	Episodic	241	86.1%
	Continuous	39	13.9%
Pain radiates to the surrounding area	Yes	197	70.4%
	No	83	29.6%
Pain worse when chewing and eating	Yes	223	79.6%
	No	57	20.4%
Eating or drinking something cold makes pain worse	Yes	188	67.1%
	No	92	32.9%
Gums swollen	Yes	101	36.1%
	No	179	63.9%
Painful tooth feels like it is loose	Yes	77	27.5%
	No	203	72.5%
Difficulty to swallow	Yes	31	11.1%
	No	249	88.9%
Painful tooth feels like it is sticking out	Yes	63	22.5%
	No	217	77.5%
Difficulty in sleeping	Yes	126	45%
	No	154	55%

Self-care and formal-care strategies for dental pain relief

For the individuals that experienced acute dental pain during the lockdown, another questionnaire was used to determine strategies for self-care and formal care to relieve dental pain (Table 3). 

**Table 3:** Self-care and formal-care strategies for pain relief for most recent toothache

		n	%
Self-care strategies			
Did you take prescription drug?	Yes	65	23.2%
	No	215	76.8%
Did you take non-prescription drug?	Yes	161	57.5%
	No	119	42.5%
Did you take home remedy?	Yes	198	70.7%
	No	82	29.3%
The home remedy	Salt and warm water	99	35.4%
	Herbs or spices (cloves, ginger, garlic)	80	28.6%
	Cold or warm pads	11	3.9%
	Others	81	28.9%
Did you speak with relative, friend, or neighbor?	Yes	152	54.3%
	No	128	45.7%
Formal-care strategies			
Did you go to a dentist?	Yes	92	32.9%
	No	188	67.1%
Did you speak to a dentist?	Yes	107	38.2%
	No	173	61.8%
Did you go to emergency room?	Yes	22	7.9%
	No	258	92.1%
Did you go to a physician's office or clinic?	Yes	36	12.9%
	No	244	74.1%
Did you speak to a pharmacist?	Yes	72	25.7%
	No	208	74.3%
Did you use 'SEHATY' application or call '937' number or similar platforms?	Yes	33	11.8%
	No	247	88.2%
Was access to these services easy?	Yes	28	10%
	No	5	1.8%
Did the service connect you to a dentist?	Yes	13	4.6%
	No	20	7.1%
Did you find the service helpful?	Yes	16	5.7%
	No	17	6.1%
Do you think it is necessary to have a virtual dental clinic?	Yes	32	11.4%
	No	1	0.4%

As seen in Table 3, the majority of the participants who used self-care strategies to relieve acute dental pain during the pandemic curfew used home remedies (70%), and non-prescribed drugs (57%), while 23% used prescribed drugs, and 20.9% relied on talking to a friend or a relative. The most commonly used home remedies were salt and warm water (35.4%) and herbs and spices (28.6%). 

In relation to formal care strategies, 32.9% have been able to go to a dentist, 25.7% spoke to a pharmacist, and 7.9% went to the emergency room. Among all participants who experienced pain during the lockdown, 33 out of 280 participants (11.8%) accessed one of the telemedicine platforms "Sehaty” or called “937". Of those, 84.8% (n=28) found access to these services easy. Only 39.3% (n=13) of the individuals that used “Sehaty” were connected to a dentist. Finally, 96.9% (n=32) of the participants who had used a teledental platform reported the necessity of having a virtual dental clinic.

A significant difference between age groups was found regarding the use of “Sehaty” application during lockdown (p-value=0.004) and regarding speaking to a dentist about the pain (p-value=0.002).

As for gender, it has been found to influence the person’s choice to take home remedies (p-value=0.023) and to go to a physician’s office for help with pain (p-value=0.022). Nevertheless, when the educational level was studied, a significant difference was found for “visiting a dentist when experiencing pain” (p-value=0.031) and for “the necessity of having a virtual dental clinic” (p-value=0.01).

After adjusting for gender, age, and educational level, females were found to be less likely to take home remedies compared to males (p-value=0.042, OR=0.53), yet, they are more likely to go to a physician’s office to treat dental pain (p-value=0.029, OR=2.48). Moreover, the older age group (51 to 60 years) was less likely to take prescribed drugs to deal with dental pain during the COVID-19 pandemic (p-value=0.001) (OR=0.23) and were less likely to visit the dentist for pain (p-value=0.000) (OR=-1.458). This significant outcome was also observed in the age group between 41 to 50 years (p-value=0.041) (OR=0.44) as seen in Table 4.

**Table 4:** Logistic regression results

Question	Demographics	Category	Significance values	Coefficient	Odds Ratio
Did you go to a physician’s office?	Gender (Ref:Male)	Female	0.029*	0.91	2.48
	Age (Ref: Age 20-30)	Age 31-40	0.70		
		Age 41-50			
		Age 51-60			
	Education (Ref: Bachelor)				
		Did you take home remedy?	Gender(Ref:Male)	Female	0.042*	-0.62	0.53
			Age(Ref: Age 20-30)	Age 31-40			
				Age 41-50			
Age 51-60					
Education (Ref: Bachelor)					
	Did you take prescription drug?		Gender(Ref:Male)	Female	0.80	0.88	1.09
			Age (Ref: Age 20-30)	Age 31-40	0.95	0.28	1.02
				Age 41-50	0.71	-0.16	0.84
Age 51-60		0.001*		-1.37	0.25
Education(Ref: Bachelor)					
		Did you take non-prescription drug?	Gender(Ref:Male)	Female	0.76		
			Age(Ref: Age 20-30)	Age 31-40			
				Age 41-50			
Age 51-60					
Education(Ref: Bachelor)					
	Did you go to a dentist?		Gender	Female	0.62	-0.15	0.85
			Age Ref: Age 20-30	Age 31-40	0.97	0.01	1.01
				Age 41-50	0.04	-0.81	0.44
Age 51-60		0.0001*		-1.45	0.23
Education(Ref: Bachelor)					
		Did you use SEHATY app or call 937 or similar platform?	Gender (Ref: Male)	Female	0.90	0.05	1.05
			Age (Ref: Age 20-30)	Age 31-40	0.09		
				Age 41-50			
Age 51-60					
Education(Ref: Bachelor)					

## Discussion

The present research is a cross-sectional study done to assess self-care methods to manage acute dental pain among adults in Makkah, Saudi Arabia during the COVID-19 pandemic curfew. The results have shown that more than one-third of the participants suffered acute dental pain during the lockdown. Odontogenic pain has been proven to be common and has been associated with caries. A recent study concluded that the caries prevalence as well as decayed, missing, and filled teeth (DMFT) scores have gone up in the past few decades among the Saudi population [20]. High caries scores in Saudi have been firmly linked to dental pain which explains why 38.5% of the study participants experienced dental pain during the lockdown.

Toothache can be extremely painful; people often desperately seek any possible way to ease it. In the current study, although only 23% of the study subjects reported taking prescribed medication, 57% have taken a non-prescribed drug. This common practice is known as “self-medication” when a person consumes a medication based on self-diagnosis without advice from a health care professional. Self-medication is widely spread in Saudi. In fact, it was stated in a recent study that 50.4% of people living in Riyadh city practised self-medication and the most common reason for such practice was dental pain [21].

Another popular way to cope with dental pain is home remedies. In this investigation, 70% have responded that they have used home remedies to alleviate toothache, most commonly warm salt, water, and herbs. This finding is similar to a cross-sectional study that was done in Makkah and Medina areas, which found that traditional home remedies such as clove, clove oil, and tahini are still being used to treat dental disease and more than half of the study participants preferred home remedies before seeking professional dental treatment [22]. There is no doubt that the practice of self-medication and home remedies has expanded during the lockdown due to the fear regarding risks involved with going to a dental clinic. Dental procedures, in general, include the generation of aerosols and droplets contaminated by microorganisms which have made them a high source of infection to both the dentist and the patient [23].

In light of the rapid spread of the COVID-19 virus at the time, dental clinics in Saudi Arabia greatly reduced their running capacity, and treatment was provided depending on the severity and urgency of the cases. The Saudi Ministry of Health has also set a great effort to expand its telemedicine portals: E-health smartphone applications and the Medical Consultation Call Center which reportedly served an estimated two million users per month [24]. Unfortunately, teledental platform consultations were not preferred among the participants of this study, where only 11% (n=33) of participants used teledentistry (Sehaty application). This finding is suggestively influenced by the unpopularity of telemedicine among the Saudi community. A study was conducted at King Abdul-Aziz University in Jeddah to evaluate the application of teledentistry in Saudi, in which around 150 dentists participated. However, only 28.3% of these dentists had known the term “teledentistry” [25]. Similarly, a 2020 research study found that 8.09% of participants utilized teledental services during the COVID-19 pandemic; the main reasons reported for underutilization of teledental services were mainly related to the necessity of technology, individuals who were indifferent and did not care enough to seek dental services, and the fact that most cases treated at the time were urgent and needed face-to-face dental care [26]. Another study stated that most patients believe that satisfactory dental care is linked to the dentist’s presence, in addition to the high cost of teledental infrastructure, the time needed to learn about these services, and the resistance to technology [27].

The current research found that older age groups were less likely to go to the dentist during the COVID-19 pandemic, this can be explained by the fact that the elderly are the most vulnerable to COVID-19. This group is at greater risk to bear different medical comorbidities which weaken their immune systems and therefore, were advised not to attend hospitals unless the condition was urgent; this led to anxiety and fear, and self-isolation [28]. A study done in Sweden on dental clinic attendance of ages 65 and above concluded that there was a significant correlation between increased age and decreased dental visits. The reasons were linked to many factors such as the physical well-being of these individuals, their mental status as well as their accessibility to dental care [29]. On the other hand, it was noted that females were more likely to visit a physician's office, which is consistent with other studies [30]. 

Teledentistry proved to be an easy alternative to receive consultation when it is hard to reach a dental clinic during lockdown [23]. In times when face-to-face interactions can impose a threat of being infected with a virus like the coronavirus, receiving dental treatment becomes hard, which in turn made the need for the implementation of teledentistry much more evident [24]. In the current study, 38.5% of the participants experienced dental pain but only 92 out of 188 were able to visit a dentist at the time of the pandemic. In such trying times, teledentistry should be accessible to everybody. Therefore, plans for the implementation, promotion, and utilization of teledental platforms, and the training of dental professionals for the provision of services through these platforms need to be considered. This might help to improve dental services by reducing the appointment load on clinics, saving resources, and setting priority for urgent cases especially when dental care is hard to access.

This study has some limitations as it is a cross-sectional study, thus the findings cannot be generalized. The questionnaire was distributed on many social media platforms which may result in selection bias and may not cover all of the intended samples. Including a larger sample size may reduce the variation and bias among the participants. However, the spread of social media users among different socioeconomic statuses is widely documented. Furthermore, recall bias could be an issue.

Our sample of patients showed a tendency to self-manage pain as 70% of the people who experienced pain reported the use of home remedies. In these cases, the availability of teledentistry can help improve self-care with the appropriate line of management until a dental visit is possible.

In the context of any pandemic with social and economic implications, access to care difficulties, and reduced time required for triage, teledentistry offers a major advantage in the dental management of patients while eliminating the previously mentioned risks.

Based on the findings of this study, it is important to increase awareness about maintaining oral health by visiting the dentist for routine and regular dental check-ups, not only when a toothache is experienced but also to seek medical advice before taking any medications. 

## Conclusions

The practice of self-medication for acute dental pain was common among the participants of this study during the COVID-19 lockdown. Moreover, teledental platform services were not commonly preferred for the diagnosis and treatment of dental pain. Therefore, it is important to educate the community and spread awareness on the proper way to deal with dental pain, especially when accessibility to professional dental treatment is difficult. Moreover, teledental services and remote dental consultations can improve service delivery, therefore, the implementation and promotion of such services need to be considered.
